# Coblator adenoidectomy in pediatric patients: a state-of-the-art review

**DOI:** 10.1007/s00405-023-08094-7

**Published:** 2023-07-26

**Authors:** Christian Calvo-Henriquez, María RuedaFernandez-Rueda, Ainhoa Garcia-Lliberos, Byron Maldonado-Alvarado, Xenia Mota-Rojas, Antonino Maniaci, Giannicola Iannella, Ignacio Jimenez-Huerta

**Affiliations:** 1 Rhinology Study Group of the Young-Otolaryngologists of the International Federations of Oto-Rhino-Laryngological Societies (YO-IFOS), Paris, France; 2 Service of Otolaryngology, Hospital Complex of Santiago de Compostela, Santiago de Compostela, Spain; 3 Service of Otolaryngology, Hospital 12 de Octubre, Madrid, Spain; 4 Service of Otolaryngology, Valencia University Hospital, Valencia, Spain; 5 Service of Otolaryngology, Hospital Virgen de los Lirios, Alcoy, Spain; 6 Service of Otolaryngology, El Bierzo Hospital, Ponferrada, Spain; 7 Department of Medical and Surgical Sciences and Advanced Technologies ‘‘GF Ingrassia’’ ENT Section, University of Catania, 95123 Catania, Italy; 8 Department of ‘Organi di Senso’, University ‘‘Sapienza’’, Viale Dell’Università, 33, 00185 Rome, Italy; 9 Master degree in rhinology and skull base, Universidad Internacional de Andalucía, Seville, Spain

**Keywords:** Adenoidectomy, Adenotonsillectomy, Coblation, Coblator, Power-assisted adenoidectomy

## Abstract

**Introduction:**

Adenoid hypertrophy is one of the main causes of nasal obstruction in ‘children. Adenoid hypertrophy can be approached either with nasal corticosteroids, or surgically when medical treatment fails. Different adenoidectomy techniques have been proposed to reduce morbidity and surgical risks, with a consequent marked increase in the use of new surgical procedures in recent years, with a progressive increase in the use of coblation. This state-of-the-art review aims to systematically review the current literature on the role of coblation in adenoidectomy.

**Methods:**

The selection criteria included children submitted to adenoidectomy with coblator vs other techniques. 11 research questions were defined. 4 databases were explored by four authors: PubMed (Medline), the Cochrane Library, EMBASE and SciELO. The level of evidence and quality of the selected articles were assessed according to assessed according to the Quality Assessment Checklist of the National Institute for Health and Clinical Excellence.

**Results:**

20 studies met the inclusion criteria: 2 metanalysis, 12 randomized clinical trial, 2 non-randomized clinical trial, 1 prospective cohort study, and 3 retrospective cohort study. It encompassed a total population of 8375 participants. Regarding the different surgical techniques, 18 studies (excluding metanalysis) performed coblation (*n* = 1550), 6 microdebridement (*n* = 883), 15 curettage (*n* = 4016), and 1 suction coagulation (*n* = 1926).

**Conclusion:**

Coblator adenoidectomy appears to offer better adenoid control compared to curettage, with a possible, although not confirmed lower rate of revision surgery. Similarly, this greater resection of adenoid tissue seems to be related to a greater reduction of nasal obstruction. The advantages of this technique are mainly less surgical bleeding—although it is not clear this is a clinically relevant difference, and less postoperative pain compared to cold curettage. The difference in pain is small, as adenoidectomy is not a painful surgery in general. There is little evidence on the control of OME and comparison with other techniques such as microdebrider adenoidectomy.

**Supplementary Information:**

The online version contains supplementary material available at 10.1007/s00405-023-08094-7.

## Introduction

Adenoid hypertrophy (AH) has been described as a natural response to increased immunologic activity in early life [1], being one of the main causes of nasal obstruction in children [2]. Furthermore, it has been identified as an underlying factor in recurrent or persistent otitis media, sinusitis, and obstructive sleep apnea (OSA). It is well known that the outcomes of upper airway dysfunction should not be taken lightly as significant upper airway disorders may lead to other severe conditions such as altered craniofacial growth and cognitive impairment.

AH can be approached either with nasal corticosteroids [3], or surgically when medical treatment fails. Adenoidectomy is one of the most common surgical procedures in pediatric patients [4], whether performed alone or associated with other surgeries. This procedure has been reported to be as high as 65 per 10,000 children in England and 50 per 10,000 children in the United States. [5]

According to the American Academy of Otolaryngology & Head and Neck Surgery (AAOHNS) the indications for adenoidectomy are four or more episodes of recurrent suppurative rhinorrhea; sleep disorders with nasal breathing obstruction; hyponasality; Otitis media with tympanic effusion for more than 3 months; malocclusion or orofacial growth disorder; cardiopulmonary complications associated with upper airway obstruction, recurrent acute and chronic otitis media with tympanic effusion. [6]

Different adenoidectomy techniques have been proposed to reduce morbidity and surgical risks, with a consequent marked increase in the use of new surgical procedures in recent years. While the most commonly used technique was cold curettage, in 2007, this trend began to be observed in a survey reporting an increase in the use of electrocautery (26%), microdebrider (20%) and coblation (7%) [7]. There is no more recent data, and these numbers may be currently different.

Coblation or ‘controlled ablation’ was first described in 2001 [8], although its use in adenoidectomy began in 2005 [9]. Coblation adenoidectomy has gained increasing attention among otolaryngologists, as it only heats up to 60 °C, causing minimal damage to the surrounding tissue.

The aim of this state-of-the-art review is to systematically review the current literature on the role of coblation in adenoidectomy, including measurable changes and side effects to guide current practice, as well as to identify knowledge gaps to conduct future research.

## Methods

This review was conducted in accordance with PRISMA guidelines. In addition, a formal PROSPERO protocol was published according to the NHS International Prospective Register of Systematic Reviews prior to the start of the study. The recommendations of the AMSTAR-2 guidelines were also followed.

### Literature search: inclusion and exclusion criteria

The criteria for considering studies for this systematic review were based on the population, intervention, comparison, and outcome framework (PICOTS).

Participants: Children

Intervention: adenoidectomy

Comparison: coblator vs other techniques

Outcomes: any measurable variable attributable to adenoidectomy. Consequently, 11 research questions were defined (1) is coblator adenoidectomy in pediatric patients associated with less intraoperative bleeding? (2) is coblator adenoidectomy in pediatric patients associated with higher operative time? (3) is coblator adenoidectomy in pediatric patients associated to less residual adenoid or postoperative recurrence? (4) is coblator adenoidectomy in pediatric patients associated to less postoperative bleeding? (5) is coblator adenoidectomy in pediatric patients associated to less postoperative pain? (6) is coblator adenoidectomy in pediatric patients associated to improvement in mucociliary clearance? (7) is coblator adenoidectomy in pediatric patients associated to improvement in rhinomanometry? (8) is coblator adenoidectomy in pediatric patients associated to improvement in eustachian tube function? (9) is coblator adenoidectomy in pediatric patients associated to higher cost? (10) is coblator adenoidectomy in pediatric patients associated to less revision rate? and (11) is coblator adenoidectomy in pediatric patients associated to more improvement in sleep disordered breathing?

Timing and setting: without limitation

Types of studies: clinical trials and prospective and retrospective cohort studies published in peer-reviewed journals. Case reports, case series, theses, narrative reviews and meeting communications were not included. There were no restrictions by date or publication type, and the research was last updated in December 2022. Studies published in English, Spanish, German, French, Italian, Portuguese, and Spanish were included.

Exclusion criteria consisted of (1) studies conducted in syndromic patients; (2) dual publications; (3) mixing of surgical techniques without subgroup analysis (4) mixing of pediatric patients (< 18 years) with adults without subgroup analysis (5) simultaneous performance of tonsillectomy without analysis of variables directly attributed to adenoidectomy and (6) less than 20 cases with coblator.

### Search strategy

We followed the recommendations of the PRISMA statement for a systematic review and searched the following databases: PubMed (Medline), the Cochrane Library, EMBASE and SciELO. A predefined search strategy was used: (“Adenoid*” OR “adenoidectomy” OR “adenotonsillectomy” OR “pharyngeal tonsil”) AND (“coblat*” OR “plasma” OR “bipolar radiofrequency” OR endoscopic).

Abstracts of retrieved articles were thoroughly reviewed by four authors, members of the YO-IFOS rhinology study group (CCH, BMA, XMR, MFR), and those potentially meeting the inclusion criteria were selected for full-text review. In case of discrepancies between reviewers regarding the selection of abstracts, the corresponding articles were included in the full-text review phase for final assessment. The references of all selected articles were also manually reviewed to identify any potentially missing publications.

### Study extraction, categorization, and analysis

Four authors (CCH, BMA, XMR, MFR) independently analyzed articles meeting the inclusion criteria extracting relevant data. Discrepancies were resolved by discussion. Variables extracted encompassed: sample size, age, surgical indication, surgical technique, and any measurable outcomes attributed to adenoidectomy.

### Assessment of study quality

The level of evidence and quality of the selected articles were assessed. The level of evidence was graded according to the Oxford Centre for Evidence-Based Medicine levels. Risk of bias was assessed according to the Quality Assessment Checklist of the National Institute for Health and Clinical Excellence: appendix F for quasi-experimental studies and case series; appendix C for randomized clinical trials.

### Statistical analysis

Data were analyzed with STATA for Macintosh v. 15.1 (StataCorp^®^). No statistical comparisons were made. STATA was used to perform mathematical analysis of mean sample size and mean age. Statistical significance level was considered at a *P* value < 0.05.

## Results

### Search results

The initial search retrieved 277 publications. After reading all titles and abstracts, 41 studies were selected for full text review. A total of 20 studies met the inclusion criteria.

6 authors were contacted twice with the aim of obtaining missing data; 1 of them responded.

Of the papers selected for full-text reading, 21 publications were excluded for the following reasons: four did not perform coblation adenoidectomy; two presented mixed techniques without subgroup analysis; two had a too small sample size; three due to study design; three did not report any measurable variable attributed to adenoidectomy; three performed simultaneous tonsillectomy without analyzing variables attributed to adenoidectomy; two mixed adult patients and children; one due to language; one was a duplicated study. References in supplementary file 1.

### Results of the included studies

A summary of the selected studies is represented in supplementary file 2 and Table 1.

**Table 1:** Summary of the evidence

Question 1: is coblator adenoidectomy in pediatric patients associated with less intraoperative bleeding?	
Available evidence	Favoring: 10 RCT (Hapalia, Balasubramanian, Shapiro, El Tahan, Singh, Mularczyk, Ozkiris, Di Rienzo, Gülšen, Chauhan); 2 NRCT (salam, Bidaye); 1 prospective cohort (Kim); 1 retrospective cohort (Gul) Against: No difference: 1 metanalysis (Sun)
Level of evidence	1a
Conclusions	Probably coblator is associated with less bleeding, despite it could not be demonstrated in a network metanalysis because of the wide confidence intervals and heterogeneity of studies
Question 2: is coblator adenoidectomy in pediatric patients associated to less postoperative bleeding?	
Available evidence	Favoring: 1 RCT (singh), 1 prospective cohort (kim -microdebrider-) Against: No difference:
Level of evidence	1b
Conclusions	Coblator is associated with less postoperative bleeding compared to microdebrider adenoidectomy. There is no evidence compared against curette
Question 3: is coblator adenoidectomy in pediatric patients associated with higher operative time?	
Available evidence	Favoring: 8 RCT (Hapalia, Ozkiris, balasubramanian, El Tahan, Singh, Mularczyk, Gülšen, Chauhan); 2 NRCT (Salam, Bidaye), 1 retrospective cohort (Gul) Against: 1 RCT (Shapiro); 1 prospective cohort (Kim -microdebrider-) No difference: 1 metanalysis (Ya-Lei Sun); 1 retrospective cohort (Sjogren)
Level of evidence	1a
Conclusions	Probably coblator is associated with longer operative time, despite it could not be demonstrated in a network metanalysis because of the wide confidence intervals and heterogeneity of studies
Question 4: is coblator adenoidectomy in pediatric patients associated to less residual adenoid or postoperative recurrence?	
Available evidence	Favoring: 8 RCT (Hapalia, balasubramanian, Di Rienzo, El Tahan; Gülšen, Chauhan, Bhat, Huang); 2 NRCT (Salam, Bidaye); 1 retrospective cohort (Gul) Against: No difference: 1 metanalysis (Ya-Lei Sun)
Level of evidence	1a
Conclusions	Probably coblator is associated with less residual adenoid, despite it could not be demonstrated in a network metanalysis because of the wide confidence intervals and heterogeneity of studies
Question 5: is coblator adenoidectomy in pediatric patients associated to less revision rate?	
Available evidence	Favoring: 1 retrospective cohort (Sjogren) Against: 1 retrospective cohort (Bhandari) No difference: 1 metanalysis (Lee)
Level of evidence	1a
Conclusions	Coblation is not associated with less revision rate compared to other adenoidectomy techniques
Question 6: is coblator adenoidectomy in pediatric patients associated to less postoperative pain?	
Available evidence	Favoring: 1 metanalysis (Ya-Lei Sun); 5 RCT (Hapalia, Singh, Mularczyk, Gülšen, Chauhan); 1 1 NRCT (Bidaye) Against: No difference: 1 NRCT (Salam); 2 RCT (Shapiro, El Tahan)
Level of evidence	1a
Conclusions	Coblator is associated with less postoperative pain in the first days after surgery compared against microdebrider, curette and suction coagulation
Question 7: is coblator adenoidectomy in pediatric patients associated to improvement in rhinomanometry?	
Available evidence	Favoring: 2 RCT (Di Rienzo, Huang) Against: No difference:
Level of evidence	1b
Conclusions	Coblator adenoidectomy decreases more nasal obstruction compared to cold curette
Question 8: is coblator adenoidectomy in pediatric patients associated to improvement in mucociliary clearance?	
Available evidence	Favoring: 1 RCT (Ozkiris) Against: No difference:
Level of evidence	2b
Conclusions	Coblator adenoidectomy increases more mucociliary clearance compared against curette
Question 9: is coblator adenoidectomy in pediatric patients associated to improvement in eustachian tube function?	
Available evidence	Favoring: 1 RCT (Gülšen) Against: No difference: 1 RCT (Bhat, Huang), 1 NRCT (Salam)
Level of evidence	1b
Conclusions	The chosen sample is not representative of OME patients. There is no evidence in this regard. There is not enough evidence regarding eustachian tube function
Question 10: is coblator adenoidectomy in pediatric patients associated to more improvement in sleep disordered breathing?	
Available evidence	Favoring: Against: No difference: 1 RCT (Huang)
Level of evidence	2b
Conclusions	There is no evidence to suggest that coblation adenoidectomy is associated with more improvement of sleep disordered breathing compared against other adenoidectomy techniques
Question 11: is coblator adenoidectomy in pediatric patients associated to higher cost?	
Available evidence	Favoring: 1 retrospective cohort (Sjogren) Against: No difference:
Level of evidence	2b
Conclusions	Coblator is more expensive than curette regarding direct costs. There is no evidence regarding indirect costs

The search strategy retrieved 20 studies, 2 metanalysis [10, 11]; 12 randomized clinical trial [12–23]; 2 non-randomized clinical trial [24, 25]; 1 prospective cohort study [26]; 3 retrospective cohort study [27–29].

The review, excluding the 2 metanalysis, encompassed a total population of 8375 participants. The mean sample size per study was 465.28 patients. The largest sample size was reported by Bhandari et al [28] (*n* = 5659), and the smallest (*n* = 40) by Di Rienzo et al. [22] and Hapalia et al. [13]

Regarding the different surgical techniques, excluding the two meta-analyses, 18 studies performed coblation (*n* = 1550), 6 microdebridement (*n* = 883), 15 curettage (*n* = 4016), and 1 suction coagulation (*n* = 1926).

Only 13 of the 20 selected studies provided their mean age. Given this limitation, the mean age adjusted for sample size was 6.37 years, with the youngest mean age being 4.58 years by Mularczyk et al [18] (microdebrider cohort) and the oldest 8.4 years, by Di Rienzo et al [22] (curette cohort).

The mean follow-up adjusted by sample size was 96.39 days, being 730 days the longest follow-up (Bhat et al.) [15], and 1 day the shortest (Bidaye et al.) [25].

### Quality of included studies: publication bias and small study bias

The risk of bias, assessed according to the National Institute for Health and Clinical Excellence´s Quality Assessment of case series studies checklist, is summarized in Table 2 for cohort studies and in Table 3 for randomized clinical trials.

**Table 2:** Assessment of the risk of bias quasi-experimental and cohort study

	Kim	Gul	Bhandari	Sjogren
1.1	+ +	+ +	+ +	+ +
1.2	+ +	+ +	+ +	+ +
1.3	+ +	+ +	+ +	+ +
2.2	+ +	+ +	+	+ +
2.4	–	–	NR	–
2.5	+ +	+ +	+ +	+ +
2.8	+ +	+	NR	NR
2.9	+ +	+ +	+ +	+ +
3.1	+ +	+ +	+ +	+ +
3.2	+ +	+ +	+ +	+ +
3.3	+	+	+	+ +
3.4	+ +	+ +	+ +	+ +
4.1	+	+ +	+ +	+ +
4.3	–	–	+ +	+ +
4.4	–	–	–	–
4.5	+ +	+	+ +	+ +
4.6	–	–	–	–
5.1			+	+
5.2	+		+	+

*NA* not applicable, *NR* not reported, +  + Well covered, + Adequately addressed, – Poorly addressed, *1.1* Is the source population or source area well described?, *1.2* Is the eligible population or area representative of the source population or area?, *1.3* Do the selected participants or areas represent the eligible population or area?, *2.2* Were interventions well described and appropriate?, *2.4* Were participants or investigators blind to exposure and comparison?, *2.5* Was the exposure to the intervention and comparison adequate?, *2.8* Were all participants accounted for at study conclusion?, *2.9* Did the setting reflect usual practice?, *3.1* Were outcome measures reliable?, *3.2* Were all outcome measurements complete?, *3.3* Were all important outcomes assessed?, *3.4* Were outcomes relevant?, *4.1* Were exposure and comparison groups similar at baseline? If not, were these adjusted?, *4.3* Was the study sufficiently powered to detect an intervention effect (if one exists)?, *4.4* Were the estimates of effect size given or calculable?, *4.5* Were the analytical methods appropriate?, *4.6* Was the precision of intervention effects given or calculable?, *5.1* Are the study results internally valid (i.e., unbiased)?, *5.2* Are the findings generalizable to the source population (i.e., externally valid)?

**Table 3:** Assessment of the risk of bias for clinical trial

	Sing	Hapalia	Huang	Bhat	Gulsen	Chauhan	Mularczycs	el tahan	Balasubramanian	Ozkiris	di rienzo	Shapiro	Salam	Bidaye
A1	+ +	+	+	+	+ +		+ +	+	+	+ +	+ +		+	
A2	+	–	–	–	+ +		+	–	–	+	+		–	
A3	+ +	–	+ +	+ +	+ +		+ +	+ +	–	+ +	+		+	
B1	+ +	+ +	+ +	+ +	+ +		+ +	+ +	+	+ +	+ +		+ +	
B2	+ +	–	–	–	–		+ +	–	–	+ +	+ +		–	
B3	–	–	–	–	–		–	–	– /NR	–	–		–	
C1	+ +	+	+ +	+ +	+ +		+ +	+ +	–	+ +	+ +		+	
C2	+ +	+	+	+	+ +		+ +	+	+	+ +	+ +		+	
C3	+ +	+ +	+ +	+ +	+ +		+ +	+ +	–	+ +	+ +		+ +	
D1	+ +	+	+ +	+ +	+		+ +	+ +	– / +	+ +	+ +		+ +	
D2	+ +	+ +	+	+	+ +		+ +	+ +	–	+ +	+ +		+ +	
D3	+ +	+	+	+	+ +		+ +	+	–	+ +	+ +		+	
D4	–	–	–	–	–		–	–	–	–	–		–	
D5	–	–	–	–	–		–	–	–	–	–		–	

*NA* not applicable, *NR* not reported, +  + Well covered, + Adequately addressed, – Poorly addressed, *A1* An appropriate method of randomization was used to allocate participants to treatment groups (which would have balanced any confounding factors equally across groups), *A2* There was adequate concealment of allocation (such that investigators, clinicians and participants cannot influence enrolment or treatment allocation), *A3* The groups were comparable at baseline, including all major confounding and prognostic factors, *B1* The comparison groups received the same care apart from the intervention(s) studied, *B2* Participants receiving care were kept 'blind' to treatment allocation, *B3* Individuals administering care were kept 'blind' to treatment allocation, *C1* All groups were followed up for an equal length of time (or analysis was adjusted to allow for differences in length of follow-up), *C2* The groups were comparable for treatment completion (that is, there were no important or systematic differences between groups in terms of those who did not complete treatment), *C3* The groups were comparable with respect to the availability of outcome data (that is, there were no important or systematic differences between groups in terms of those for whom outcome data were not available), *D1* The study had an appropriate length of follow-up, *D2* The study used a precise definition of outcome, *D3* A valid and reliable method was used to determine the outcome, *D4* Investigators were kept 'blind' to participants' exposure to the intervention, *D5* Investigators were kept 'blind' to other important confounding and prognostic factors

## Discussion

Interest in coblation adenoidectomy has grown considerably in recent times, as reflected in the literature, where most of the evidence has been published in the last 10 years.

Two previous systematic reviews and meta-analyses have been performed with the aim of assessing the potential benefits of coblation adenoidectomy compared to other techniques [10, 30]. One of them (Aleem et al.) [30] was not included in our study as it mixed adults with children, and coblation with other endoscopic techniques (5 of the 14 included studies did not perform coblation). The other study (Sun et al.) [10] is a high quality review and network meta-analysis comparing different adenoidectomy techniques assessing pain, blood loss, intraoperative time and postoperative residual tissue. The main problem with this study was the heterogeneity in the methods used to assess blood loss and intraoperative time, which could make comparison between groups inappropriate.

The available evidence is still scarce in the literature and most publications are focused on the analysis of operative time and intraoperative bleeding. However, operative time and bleeding are not the most relevant variables for otolaryngologists when selecting one technique over another [7], but rather those that have to do with clinical outcomes.

### Question 1: is coblator adenoidectomy in pediatric patients associated with less intraoperative bleeding?

Regarding intraoperative bleeding, 10 RCTs [12, 13, 16–23] found differences in favor of coblator. However, the network meta-analysis by Sun et al [10] could not identify any statistically significant difference given the wide Odds ratio confidence interval. This lack of statistical significance may reflect the heterogeneity of methods to measure bleeding. In addition, the clinical relevance of this difference remains debatable and, despite being statistically significant, the difference between groups is small and therefore may not be clinically important.

In conclusion, coblator may be associated with less bleeding, although this could not be demonstrated in a network meta-analysis due to wide confidence intervals and heterogeneity of studies.

### Question 2: is coblator adenoidectomy in pediatric patients associated to less postoperative bleeding?

In this respect, evidence is scarce with only one RCT [12] and one cohort study [26] found in the literature. Both studies compare adenoidectomy with coblator with adenoidectomy with microdebrider. The available evidence suggests less postoperative bleeding in the coblator group. For studies comparing coblator and curettage, there is no analysis comparing the two groups.

### Question 3: is coblator adenoidectomy in pediatric patients associated with higher operative time?

This is a relevant point considering that a previous study found that one of the most significant factors contributing to the choice of instrument in adenotonsillectomy is the duration of the procedure [31]. 

The literature suggests that coblator adenoidectomy is associated with longer operative time compared to cold curettage, however, it is not superior to other power-assisted techniques such as the microdebrider. Thus, the pooled data from the network meta-analysis by Sun et al [10] could not identify any statistically significant differences.

The main difficulty in comparing studies is that different authors report operative time differently. Thus, some groups calculate the total time spent in the operating theatre or operating room, others calculate it as the time from induction of anesthesia to the end of surgery [19], others until the patient wakes up or until hemostasis is completed, [12, 23]; while others do not explain it clearly [21, 27]. 

Shapiro et al [23], who were the only group to find less operative time compared to cold dissection, in their study they measured operative time including hemostasis, which could justify this difference.

In conclusion, coblator may be associated with longer operative time, although this could not be demonstrated in a network meta-analysis due to wide confidence intervals and heterogeneity of studies.

### Question 4: is coblator adenoidectomy in pediatric patients associated to less residual adenoid?

A distinction must be made between simple adenoid persistence, which ranges between 1.3 and 26% [32], and reintervention, which ranges between 0.5 and 2% [33]. Adenoid persistence, although not important per se, is relevant, as persistence of adenoid tissue at 1 month after surgery has been related to adenoid regrowth at 1-year follow-up. Therefore, the persistence of some degree of adenoid tissue after surgery is of importance, and surgery should aim to remove as much adenoid tissue as possible [26]. In terms of what we found in the evidence, all studies, with the exception of Sun et al. meta-analysis [10], found fewer residual adenoids in the coblator group. The Sun et al. network meta-analysis [10] did find more residual adenoid tissue in the curette group compared to the coblator group, although this difference was not statistically significant.

In conclusion, coblator is probably associated with less residual adenoids, although this could not be demonstrated in a network meta-analysis due to the wide confidence intervals and heterogeneity of the studies.

### Question 5: is coblator adenoidectomy in pediatric patients associated to less revision rate?

A different aspect to symptoms or recurrence (question 4), is reintervention. Not all patients with adenoid recurrence have symptoms severe enough to justify surgical reintervention. Some authors have hypothesized that coblation may create fibrosis in the lamina propria and less lymphocyte infiltrate, which may be related to a more stable and definitive control of adenoid hypertrophy [22]. However, the only meta-analysis (Lee et al.) [11] that evaluated the revision rate according to different adenoidectomy techniques could not identify any significant difference between excisional methods.

In conclusion, coblation is not associated with a lower revision rate compared to other adenoidectomy techniques.

### Question 6: is coblator adenoidectomy in pediatric patients associated to less postoperative pain?

Another variable that could justify the selection of one technique over another is pain. A total of 10 studies analyzed pain intensity [10, 12, 13, 16–19, 23–25], including one meta-analysis [10]. Sun et al. network meta-analysis [10] reported significantly lower pain in the coblator group compared to the cold curette group, mixing VAS score results (mean difference = 3.45, 95% CI [0.95, 6.01]).

The available evidence is strong. Coblation was associated with less pain compared to cold curettage in the first days after surgery [10, 12, 13, 16–18, 25]. However, no difference was found at 1 week, [16] results that are similar when compared to the microdebrider technique. [12]

This difference in pain intensity is statistically significant. However, this difference is mild, therefore, it is not clear its clinical relevance.

### Question 7: is coblator adenoidectomy in pediatric patients associated with greater improvement in nasal obstruction?

There are no studies using quality of life surveys such as sinus and nasal quality of life questionnaires. Regarding symptoms, there is only one study with rhinomanometry [22], with better results for coblator compared to curette. Rhinomanometry, with and without decongestant, is a good diagnostic tool that has been proposed to select children for adenoidectomy. [34]

The other study, by Huang et al. [14] only assessed VAS score, but the improvement was also more noticeable in the coblator cohort compared to curette.

In conclusion, the available evidence is scarce. It suggests that coblator adenoidectomy is associated with greater improvement in nasal obstruction compared to cold curette.

### Question 8: is coblator adenoidectomy in pediatric patients associated to improvement in mucociliary clearance?

Only one group studied mucociliary transport time (MCTT) [21]. For this purpose, they used scintigraphy and observed that coblation adenoidectomy improved MCTT, whereas cold curette did not.

The justification for this difference could be that adenoid hypertrophy is associated with rhinitis [35]. Coblator adenoidectomy may solve better the rhinitis, improving MCTT. Another explanation may be nasal obstruction associated with adenoid hypertrophy. Lack of nasal breathing has been found to be a detrimental factor in MCTT. Therefore, improvement in MCTT may reflect improvement in nasal breathing, which has been assessed in question 7, and coblator seems to offer better results than curette.

In conclusion, the evidence is scarce. The available evidence suggests that coblator adenoidectomy is associated with greater improvement in MCTT compared to cold curettage.

### Question 9: is coblator adenoidectomy in pediatric patients associated to improvement in eustachian tube function?

Although OME is one of the main indications for adenoidectomy, there are few studies evaluating this variable in the literature. Tubal tonsil is a frequent site of recurrence [36] and therefore, a source of persistent symptoms after adenoidectomy [36, 37]. However, there is reason to believe that coblation may offer better results in the management of OME probably justified because endoscopic vision offers better control of the tubaric tonsils.

Two of the three authors who evaluated Eustachian tube function excluded patients with OME [15, 16], so, although remarkable, their cohort is not representative of real patients. Gülsen et al [16] reported no Eustachian tube dysfunction in the first days after surgery in the coblation cohort, while 19.4% of curettage patients had Eustachian tube dysfunction in the first days after surgery, returning to normal values within one week. Bhat et al [15] reported no difference in middle ear pressure after 3 months follow-up. Again, the sample was also not representative of the real target population, patients with OME.

In conclusion, the sample chosen in the mentioned studies was not representative of patients with OME. Therefore, there is no evidence in this respect existing insufficient evidence on the Eustachian tube function in relation to the adenoidectomy technique performed.

### Question 10: is coblator adenoidectomy in pediatric patients associated with greater improvement in sleep disordered breathing?

Only one RCT [14] has been found evaluating this variable. In this study, they were unable to identify any statistically significant difference when comparing coblator adenoidectomy with adenoidectomy with curettage. Although isolated adenoidectomy may have some role in the treatment of pediatric sleep disordered breathing [38], its role is limited. Therefore, the surgical technique of adenoidectomy is not expected to influence this outcome.

### Question 11: is coblator adenoidectomy in pediatric patients associated with a higher cost?

The last important variable influencing the preferred instrument or technique is cost [31]. The equipment required for coblation is significantly more expensive than other options such as cold curettage or electrocautery. However, the cost analysis must take into account additional costs including the duration of surgery, parental days off work, use of analgesics, complication rate and revision surgery, among others. There is only one cost study [29] that describes a value of $200 more per patient compared to cold curette technique, but $35 less than the microdebrider. However, in this study only the direct costs were considered, without taking into account the additional costs mentioned above.

In conclusion, the coblator is more expensive than the curette in terms of direct costs with no evidence on indirect costs.

Although the above results could guide our decision on the choice of technique, there are several confounding factors that could impair the analysis.

Adenoid size is an important confounder. It has been reported to be related to recurrence rate [39], and postoperative pain [18] but only one author [18] have controlled this confounding factor in a stratified analysis.

Another confounding factor to consider is the surgical criteria, as most authors did not specify the surgical criteria. Surgery may be indicated for several reasons including sleep apnea, OME, chronic rhinosinusitis or nasal obstruction and results may differ according to the indication. As an example, a multivariate analysis showed that there is a significant risk of revision adenoidectomy for patients with an ear-related indication for adenoidectomy, (OR 10.8) [40]. However, of all the selected studies, only Huang et al [14] were the only ones to perform a subgroup analysis with respect to surgical indication.

Surgical technique and surgeon skills may also influence outcomes. Thus, level of training has been reported to be a factor related to the risk of revision surgery, with patients operated on by trainees being 50% more likely to require revision surgery [40]. Likewise, the extent of adenoidectomy has been reported to be the most important factor related to the recurrence rate [41].

Coblator can be utilized following different methods, as it can be used through the nose or through the mouth. As well as the way to visualize the adenoids, using a transnasal endoscope, transoral angulated endoscope or transoral mirror. It can also change the power set, according to the delivered energy. There are no studies comparing these variables.

Finally, younger age has been proposed in several studies as a factor positively related to adenoid regrowth and revision surgery, as each year decreases the risk of revision surgery by 30%. However, none of the authors have stratified their results according to age.

Another potential confounding factor not considered in this review is the concomitant tonsillectomy. In this review we have excluded studies with concomitant surgery. This sample may not reflect the common patient, as usually adenoidectomy is associated with tonsillectomy.

Coblator can be used in different ways, as it can be performed either through the nose or through the mouth. The same applies to the type of instrument used to visualize the adenoids, either using a transnasal endoscope, an angled transoral endoscope or a transoral mirror. Furthermore, the power setting may also vary depending on the energy delivered. In this respect, there are no studies comparing these variables.

Regarding to age, younger age has been proposed in several studies as a factor positively related to adenoid regrowth [39], and revision surgery, as each year decreases the risk of revision surgery by 30% [40]. However, none of the authors have stratified their results according to age in this regard either.

Another potential confounding factor not considered in this review is concomitant tonsillectomy, as studies with concomitant surgery have been excluded; consequently, it is possible that this sample does not reflect the common patient, thus adenoidectomy is usually associated with tonsillectomy.

Finally, it should be kept in mind that coblation adenoidectomy may have its own risks and disadvantages that should be measured against its benefits. The most remarkable is that it may impair the presence of enough tissue to perform histology assessment. In fact, despite the evidence is scarce, some authors have suggested that some secondary effects are more prone to occur with the coblation than microdebrider [42]. 

## Conclusion

Coblator adenoidectomy appears to offer better adenoid control compared to curettage, with a possible, although not confirmed lower rate of revision surgery. Similarly, this greater resection of adenoid tissue seems to be related to a greater reduction of nasal obstruction.

The advantages of this technique are mainly less surgical bleeding- although it is not clear this is a clinically relevant difference-, and less postoperative pain compared to cold curettage. The difference in pain is small, as adenoidectomy is not a painful surgery in general. There is little evidence on the control of OME and comparison with other techniques such as microdebrider adenoidectomy.

## Supplementary Information

Below is the link to the electronic supplementary material.

Supplementary file1 (DOCX 19 KB)

Supplementary file2 (DOCX 38 KB)

## Data Availability

Not applicable.
