# Supplementary material for: Coblator adenoidectomy in pediatric patients: a state-of-the-art review
Source: Eur Arch Otorhinolaryngol. 2023 Jul 26;280(10):4339–49. doi: 10.1007/s00405-023-08094-7 (PMC10477144; doi:10.1007/s00405-023-08094-7)
Supplement: Supplementary file 1 — Supplementary file1 (DOCX 19 KB) [file 405_2023_8094_MOESM1_ESM.docx]

**Did not performed coblation adenoidectomy (n=4)**:

- Wright ED, Manoukian JJ, Shapiro RS. Ablative adenoidectomy: a new technique using simultaneous liquefaction/aspiration. J Otolaryngol. 1997 Feb;26(1):36–43. E. Wright, J. Manoukian, R. Shapiro, Ablative adenoidectomy: a new technique using simultaneous liquefication/aspiration, J. Otolaryngol. 26 (1997) 36–43.
- Songu M, Altay C, Adibelli ZH, Adibelli H. Endoscopic-assisted versus curettage adenoidectomy: a prospective, randomized, double-blind study with objective outcome measures. Laryngoscope. 2010 Sep;120(9):1895–9.
- Mun IK, Yoo SH, Mo JH. Long-term outcome of concurrent coblator turbinoplasty with adenotonsillectomy in children with allergic rhinitis. Acta Otolaryngol. 2021 Mar;141(3):286–92.
- Elzayat S, Elsherif H, Aouf M. Trans-oral endoscopic assisted radio-frequency for adenoid ablation; A randomized prospective comparative clinical study. Auris Nasus Larynx. 2021 Aug;48(4):710–7.

**Mixed techniques without subgroup analysis (n=2)**:

- Dearking AC, Lahr BD, Kuchena A, Orvidas LJ. Factors associated with revision adenoidectomy. Otolaryngol Head Neck Surg. 2012 Jun;146(6):984–90.
- Aleem M, Rabie A, Ibrahim A. Endoscopic coblation versus cold curettage adenoidectomy. PAN Arab Journal of Rhinology. 2021 Aug 9;11(1):26–36.

**Small sample size (n=2)**:

- Grindle CR, Murray RC, Chennupati SK, Barth PC, Reilly JS. Incidence of revision adenoidectomy in children. Laryngoscope. 2011 Oct;121(10):2128–30.
- Ferreira MS, Mangussi-Gomes J, Ximendes R, Evangelista AR, Miranda EL, Garcia LB, et al. Comparison of three different adenoidectomy techniques in children - has the conventional technique been surpassed? Int J Pediatr Otorhinolaryngol. 2018 Jan;104:145–9.

**Study design (n=3)**:

- Adeyemi A, Delhougne G. PMD51 - COMPARISON OF COSTS AND OUTCOMES BETWEEN COBLATION TECHNOLOGY AND ELECTROCAUTERY FOLLOWING TONSILLECTOMY AND ADENOIDECTOMY PROCEDURES. Value in Health. 2018 Oct 1;21:S251.
- Türkoğlu Babakurban S, E E. Adenoidectomy: current approaches and review of the literature. Kulak burun bogaz ihtisas dergisi : KBB = Journal of ear, nose, and throat [Internet]. 2016 Jun [cited 2023 May 1];26(3). Available from:
- Kim SY, Lee WH, Rhee CS, Lee CH, Kim JW. Regrowth of the adenoids after coblation adenoidectomy: cephalometric analysis. Laryngoscope. 2013 Oct;123(10):2567–72.

**Not reporting any measurable variable attributed to adenoidectomy (n=3)**:

- Timms MS, Ghosh S, Roper A. Adenoidectomy with the coblator: a logical extension of radiofrequency tonsillectomy. J Laryngol Otol. 2005 May;119(5):398–9.
- Palmer JM. Bipolar radiofrequency for adenoidectomy. Otolaryngol Head Neck Surg. 2006 Aug;135(2):323–4.
- Glade RS, Pearson SE, Zalzal GH, Choi SS. Coblation adenotonsillectomy: an improvement over electrocautery technique? Otolaryngol Head Neck Surg. 2006 May;134(5):852–5.

**Performing simultaneous tonsillectomy without analyzing variables attributed to adenoidectomy (n=3)**:

- Paramasivan VK, Arumugam SV, Kameswaran M. Randomised comparative study of adenotonsillectomy by conventional and coblation method for children with obstructive sleep apnoea. Int J Pediatr Otorhinolaryngol. 2012 Jun;76(6):816–21.
- Shakeel M, Trinidade A, Al-Adhami A, Supriya M, Kubba H. Coblation adenotonsillectomy in children. J Coll Physicians Surg Pak. 2012 Sep;22(9):579–81.
- Qiao Y, Chen J. Efficacy of Low-Temperature Plasma-Assisted Unilateral/Bilateral Tonsillectomy and Adenoidectomy in Children with Obstructive Sleep Apnea Hypopnea Syndrome. Med Sci Monit. 2021 Sep 9;27:e930792.

**Mixed adult patients and children (n=2)**:

- Agrawal V, Agarwal PK, Agrawal A. Defining the Surgical Limits of Adenoidectomy so as to Prevent Recurrence of Adenoids. Indian J Otolaryngol Head Neck Surg. 2016 Jun;68(2):131–4.
- McCoy JL, Maguire RC, Tobey ABJ. Cost benefit of coblation versus electrocautery adenotonsillectomy for pediatric patients. Int J Pediatr Otorhinolaryngol. 2020 Sep;136:110197.

**Language (n=1):**

- Liu H, Liang M, Zhong-Wu S. Assessment of the intraoperative wound degree as well as postoperative immune function and ventilation function of plasma radiofrequency ablation for the treatment of adenoid hypertrophy. Journal of Hainan Medical University [Internet]. 2023 [cited 2023 May 1];3(1).

**Duplicated studies (n=1)**:^1^

- Balasubramanian T, Vrinda B, Nair. Coblation adenoidectomy our experience. Otolaryngology Online Journal. 2014 Mar 15;4:38–43.
