# Supplementary material for: Coblator adenoidectomy in pediatric patients: a state-of-the-art review
Source: Eur Arch Otorhinolaryngol. 2023 Jul 26;280(10):4339–49. doi: 10.1007/s00405-023-08094-7 (PMC10477144; doi:10.1007/s00405-023-08094-7)
Supplement: Supplementary file 2 — Supplementary file2 (DOCX 38 KB) [file 405_2023_8094_MOESM2_ESM.docx]

Supplementary table: summary of the evidence. NR (not reported). SUCRA (surface under the cumulative ranking curve). RCT (randomized clinical trial). VAS (visual analogue scale). Min (minutes). Ml (milliliters). Pa (Pascals). Mm (millimeters).

| Author (Year) | Study design and level of evidence | Surgical technique | Sample size and sex | Age (mean ± SD; range) | Reason for adenoidectomy | Follow-up (days) | main outcome | results before | | results after |
| --- | --- | --- | --- | --- | --- | --- | --- | --- | --- | --- |
| Sun YL (2022) | Network Metanalysis  Level 1a | Coblation | 771 (NR) | NR ± NR (NR) | NR | NR | Intraoperative time (SUCRA) | 0.17 | | |
|  |  |  |  |  |  |  | Bleeding (SUCRA) | 0.73 | | |
|  |  |  |  |  |  |  | Residual adenoids (SUCRA) | 0.89 | | |
|  |  | Curette | 539 (NR) | NR ± NR (NR) |  |  | Intraoperative time (SUCRA) | 0.83 | | |
|  |  |  |  |  |  |  | Bleeding (SUCRA) | 0.40 | | |
|  |  |  |  |  |  |  | Residual adenoids (SUCRA) | 0.00 | | |
|  |  | Suction diathermy | 148 (NR) | NR ± NR (NR) |  |  | Intraoperative time (SUCRA) | 0.63 | | |
|  |  |  |  |  |  |  | Bleeding (SUCRA) | 0.63 | | |
|  |  |  |  |  |  |  | Residual adenoids (SUCRA) | 0.61 | | |
|  |  | Microdebrider | 871 (NR) | NR ± NR (NR) |  |  | Intraoperative time (SUCRA) | 0.37 | | |
|  |  |  |  |  |  |  | Bleeding (SUCRA) | 0.24 | | |
|  |  |  |  |  |  |  | Residual adenoids (SUCRA) | NA | | |
| Abdul Salam RT (2021) | Non randomized clinical trial  Level 2b | Coblator | 25 (13 M; 12 F) | 6.6 ± 1.58 (4-9) | Symptoms of adenoid hypertrophy.  Adenoid hyperplasia with x-ray and/or nasal endoscopy | 90 | Intraoperative time (min) | **14.4 ± 2.58*** | | |
|  |  |  |  |  |  |  | Bleeding (ml) | **10.6 ± 4.16*** | | |
|  |  |  |  |  |  |  | Residual adenoids (%) | **24*** | | |
|  |  |  |  |  |  |  | Pain day 1 (VAS score) | 3.16 | | |
|  |  |  |  |  |  |  | Tympanogram (Jerger) (%) | A (48) / B (52) | A (100) / B (0) | |
|  |  | Curette | 25 (15 M; 10 F) | 6.16 ± 1.72 (4-9) |  |  | Intraoperative time (min) | **9.44 ± 3.56*** | | |
|  |  |  |  |  |  |  | Bleeding (ml) | **30.36 ± 6.32*** | | |
|  |  |  |  |  |  |  | Residual adenoids (%) | **80*** | | |
|  |  |  |  |  |  |  | Pain day 1 (VAS score) | 3.88 | | |
|  |  |  |  |  |  |  | Tympanogram (Jerger) (%) | A (40) / B (60) | A (92) / B (0.8) | |
| Singh J (2020) | Blinded RCT  Level 1b | Coblator | 70 (41 M; 29 F) | 6.9 ± 2.8 (NR) | <15 years.  Adenoids degree ≥ 3 (Clements) | 21 | Intraoperative time (min) | **22.04 ± 3.3*** | | |
|  |  |  |  |  |  |  |  |  |  |  |
|  |  |  |  |  |  |  | Bleeding (scale 0-5) | **1.4 ± 1.04*** | | |
|  |  |  |  |  |  |  | Pain day 1 / day 3 (VAS score) | **2.6 ± 0.99* / 1.17 ± 1.1*** | | |
|  |  | Microdebrider | 70 (47 M; 23 F) | 7.2 ± 2.3 (NR) |  |  | Intraoperative time (min) | **12.78 ± 3.18*** | | |
|  |  |  |  |  |  |  |  |  |  |  |
|  |  |  |  |  |  |  | Bleeding (scale 0-5) | **3.5 ± 0.9*** | | |
|  |  |  |  |  |  |  | Pain day 1 / day 3 (VAS score) | **7.14 ± 0.99* / 4.08 ± 1.42*** | | |
| Hapalia VB (2020) | Open RCT  Level 2b | Curette | 20 (NR) | NR ± NR (NR) | 4–17 years, complaints suggestive of adenoid enlargement and/or chronic adenoiditis. | 30 | Intraoperative time (min) | **10.3 ± 3.66*** | | |
|  |  |  |  |  |  |  | Bleeding (ml) | **28.5 ± 3.66*** | | |
|  |  |  |  |  |  |  | Residual adenoids (%) | **20*** | | |
|  |  |  |  |  |  |  | Pain (days) | **2.67 ± 0.9*** | | |
|  |  | Coblator | 20 (NR) | NR ± NR (NR) |  |  | Intraoperative time (min) | **15.55 ± 3.49*** | | |
|  |  |  |  |  |  |  | Bleeding (mL) | **19.0 ± 3.88*** | | |
|  |  |  |  |  |  |  | Residual adenoids (%) | **0*** | | |
|  |  |  |  |  |  |  | Pain (days) | **2.0 ± 0.95*** | | |
| Huang H (2019) | Open RCT  Level 2b | Curette + microwave | 100 (51M; 49 F) | 7.0 ± 2.4 (4-13) | Snoring (64); hearing loss (39); nasal congestion (82) | 180 | Snoring (n=64) (%) | Remarkably effective (43.75) / effective (48.44) / not effective (7.81) | | |
|  |  |  |  |  |  |  | Hearing loss (n=39) (%) | Remarkably effective (51.28) / effective (33.33) / not effective (15.38) | | |
|  |  |  |  |  |  |  | Nasal congestion (n=82) (%) | Remarkably effective (37.80) / effective (32.93) / not effective (29.27) | | |
|  |  |  |  |  |  |  | Residual adenoids (n=100) (%) | Remarkably effective (58.00) / effective (11.00) / not effective (31.00) | | |
|  |  | Coblator | 100 (57 M; 43 F) | 6.5 ± 2.6 (5-12) | Snoring (55); hearing loss (41); nasal congestion (70) |  | Snoring (n=55) (%) | Remarkably effective (54.55) / effective (32.73) / not effective (12.73) | | |
|  |  |  |  |  |  |  | Hearing loss (n=41) (%) | Remarkably effective (43.90) / effective (36.59) / not effective (19.51) | | |
|  |  |  |  |  |  |  |  |  |  |  |
|  |  |  |  |  |  |  | Nasal congestion (n=70) (%) |  |  |  |
|  |  |  |  |  |  |  |  | **Remarkably effective (57.14) / effective (27.14) / not effective (15.71)*** | | |
|  |  |  |  |  |  |  |  |  |  |  |
|  |  |  |  |  |  |  | Residual adenoids (n=100) (%) |  |  |  |
|  |  |  |  |  |  |  |  | **Remarkably effective (60) / effective (22) / not effective (18)*** | | |
|  |  |  |  |  |  |  |  |  |  |  |
|  |  |  |  |  |  |  |  |  |  |  |
| Bhat SN (2019) | Open RCT  Level 2b | Curette | 30 (17 M; 13 F) | NR ± NR (7-13) | NR | 730 | Adenoid size (Clemens; %) | I (6.7) / II (36.7) / III (43.3) / IV (13.3) | I (30.0) / II (26.7) / III (0.0) / IV (0.0) / none (43.3) | |
|  |  |  |  |  |  |  | Middle ear pressure (NR) | 127.5 ± NR | Day 1 (121.5 ± NR) / Day 7 (116.0 ± NR) / Day 30 (115.0 ± NR) | |
|  |  | Coblator | 30 (14 M; 16 F) | NR ± NR (7-13) |  |  | Adenoid size (Clemens; %) | I (0.0) / II (46.7) / III (40.0)/ IV (13.3) | I (13.3) / II (13.3) / III (3.3) / IV (0.0) / none (70.0) | |
|  |  |  |  |  |  |  | Middle ear pressure (NR) | 130.0 ± NR | Day 1 (121.5 ± NR) / Day 7 (119.0 ± NR) / Day 30 (111.5 ± NR) | |
| Gülšen S (2019) | Blinded RCT  Level 1b | Curette | 36 (18 M; 18 F) | 6.9 ± 3.1 (5-11) | Nasal obstruction, snoring, postnasal discharge | 30 | Bleeding (ml) | **43.7 ± 9.3*** | | |
|  |  |  |  |  |  |  | Residual adenoid (%) | **22.2*** | | |
|  |  |  |  |  |  |  | Intraoperative time (min) | **12.1 ± 5.4*** | | |
|  |  |  |  |  |  |  | Pain (Day) (VAS score) | **Day 1 (6.1 ± 2.8)* / Day 2 (4.1 ± 3.1)*** | | |
|  |  |  |  |  |  |  | Eustachian tube dysfunction (%) | **80.5*** | | |
|  |  | Coblator | 36 (18 M; 18 F) | 7.2 ± 4.5 (5-11) |  |  | Bleeding (ml) | **24.3 ± 5.1*** | | |
|  |  |  |  |  |  |  | Residual adenoid (%) | **0*** | | |
|  |  |  |  |  |  |  | Intraoperative time (min) | **25.7 ± 7.9*** | | |
|  |  |  |  |  |  |  | Pain (Day) (VAS score) | **Day 1 (3.1 ± 1.9)* / day 2 (2.3 ± 1.2)*** | | |
|  |  |  |  |  |  |  | Eustachian tube dysfunction (%) | **19.4*** | | |
| Chauhan VM (2019) | Blinded RCT  Level 2b | Curette | 38 (NR) | NR ± NR (5-11) | Nasal obstruction, mouth breathing, snoring. | 90 | Intraoperative time (min) | **11 ± NR*** | | |
|  |  |  |  |  |  |  | Bleeding (ml) | **51 ± NR*** | | |
|  |  | Coblator | 32 (NR) |  |  |  | Intraoperative time (min) | **29 ± NR*** | | |
|  |  |  |  |  |  |  | Bleeding (ml) | **23 ± NR*** | | |
| Gul F (2019) | Retrospective Cohort study  Level 2b | Coblator | 51 (31 M; 20 F) | 4.77 ± 3.18  (3-12) | NR | 486 (365-780) | Bleeding (ml) | **5.75 ± 3.6*** | | |
|  |  |  |  |  |  |  | Intraoperative time (min) | **23.3 ± 11.4*** | | |
|  |  |  |  |  |  |  | Postoperative recurrence (%) | **6.06*** | | |
|  |  | Curette | 33 (18 M; 15 F) | 5.13 ± 3.34  (3-12) |  |  | Bleeding (ml) | **11.58 ± 7.2*** | | |
|  |  |  |  |  |  |  | Intraoperative time (min) | **14.5 ± 5.1*** | | |
|  |  |  |  |  |  |  | Postoperative recurrence (%) | **15.6*** | | |
| Lee CH (2019) | Metanalysis  Level 1b | Curette | 95727 (NR) | 4.69 ± 1.62 (NR) | NR | (180-3650) | Revision surgery (%) | **0.6 ± 0.31** | | |
|  |  | Suction cautery |  |  |  |  | Revision surgery (%) | **1.6 ± 0.35** | | |
|  |  | Microdebrider |  |  |  |  | Revision surgery (%) | **2.3 ± 4.61** | | |
|  |  | Coblator |  |  |  |  | Revision surgery (%) | **1.8 ± 2.15** | | |
| Bidaye R (2019) | Non randomized clinical trial  Level 2b | Coblator | 30 (15 M; 15 F) | 6.33 ± 1.40 (6-12) | Adenoid hyperplasia with x rays. Children 6-12 year | 1 | Bleeding (ml) | **32.47 ± 8.61*** | | |
|  |  |  |  |  |  |  | Intraoperative time (min) | **18.27 ± 4.18*** | | |
|  |  |  |  |  |  |  | Residual tissue x Ray (Y:N ratio) | **0:100*** | | |
|  |  |  |  |  |  |  | Pain day 1 (VAS score) | **3.87 ± 1.17*** | | |
|  |  | Curette | 30 (19 M; 11 F) | 6.97 ± 1.87 (6-12) |  |  | Bleeding (ml) | **44.33 ± 12.68*** | | |
|  |  |  |  |  |  |  | Intraoperative time (min) | **4.17 ± 1.21*** | | |
|  |  |  |  |  |  |  | Residual tissue x Ray (Y:N ratio) | **40:60*** | | |
|  |  |  |  |  |  |  | Pain day 1 (VAS score) | **6.37 ± 1.07*** | | |
| Bhandari N (2018) | Historic cohort study  Level 2b | Microdebrider | 212 (NR) | NR ± NR (NR) | Infectious, OME, nasal obstruction, or adenoid hypertrophy with sleep disordered breathing | NR | Revision surgery (%) | **1.42*** | | |
|  |  | Coblator | 382 (NR) |  |  |  | Revision surgery(%) | **0.79*** | | |
|  |  | Suction coagulation | 1926 (NR) |  |  |  | Revision surgery(%) | **0.36*** | | |
|  |  | Curette | 3139 (NR) |  |  |  | Revision surgery(%) | **0.03*** | | |
| Sjogren PP (2017) | Retrospective Cohort study  Level 2b | Curette + electrocautery | 372 (NR) | 4.5 ± 3.7 (NR) | Adenoid hypertrophy (n=822), chronic adenoiditis (n=152), sinusitis (n=38), chronic rhinitis (n=24), otitis media (n=20), obstructive sleep apnea (n=9) | 21 | Cost ($) | **597*** | | |
|  |  |  |  |  |  |  | Surgical time (min) | 24.7 ± 8.1 | | |
|  |  |  |  |  |  |  | Revision rate (%) | **2.7*** | | |
|  |  | Microdebrider | 278 (NR) |  |  |  | Cost ($) | **833*** | | |
|  |  |  |  |  |  |  | Surgical time (min) | 28.7 ± 11.00 | | |
|  |  |  |  |  |  |  | Revision rate (%) | **9.7*** | | |
|  |  | Coblator | 415 (NR) |  |  |  | Cost ($) | **797*** | | |
|  |  |  |  |  |  |  | Surgical time (min) | 26.2 ± 9.8 | | |
|  |  |  |  |  |  |  | Revision rate (%) | **5.3*** | | |
| Mularczyk C (2017) | Blind RCT  Level 1b | Coblator | 50 (NR) | 4.96 ± 3.12 (NR) | NR | 3 | Operative time (min) | **5.50 ± 2.07*** | | |
|  |  |  |  |  |  |  | Pain (days) | **1.53 ± 1.03*** | | |
|  |  |  |  |  |  |  | Bleeding (ml) | **0 (92%); <10 (6%); 10-20 (2%); 20-50 (0%)*** | | |
|  |  | Microdebrider | 51 (NR) | 4.58 ± 3.03 (NR) |  |  | Operative time (min) | **9.47 ± 3.98*** | | |
|  |  |  |  |  |  |  | Pain (days) | **2.05 ± 1.12*** | | |
|  |  |  |  |  |  |  | Bleeding (ml) | **0 (2%); <10 (64.7%); 10-20 (29.4%); 20-50 (3.9%)*** | | |
| El Tahan AR (2016) | RCT  Level 2b | Coblator | 100 (51 M; 49 F) | 6.0 ± 1.33 (3-10) | Chronic nasal obstruction and discharge, with symptoms and signs of adenoid hypertrophy | 365 | Operative time (min) | **15.0 ± 1.38*** | | |
|  |  |  |  |  |  |  | Bleeding (ml) | **10.0 ± 2.58*** | | |
|  |  |  |  |  |  |  | Pain (VAS day 1; day 7) | 4.46 ± 0.50 / 1.55 ± 0.50 | | |
|  |  |  |  |  |  |  | Postoperative recurrence (%) | **2*** | | |
|  |  | Curette | 100 (52 M; 48 F) | 6.0 ± 1.28 (3-10) |  |  | Operative time (min) | **10.0 ± 1.15*** | | |
|  |  |  |  |  |  |  | Bleeding (ml) | **25.0 ± 2.92*** | | |
|  |  |  |  |  |  |  | Pain (VAS day 1 / day 7) | 4.52 ± 0.50 / 1.51 ± 0.50 | | |
|  |  |  |  |  |  |  | Postoperative recurrence (%) | **10*** | | |
| Balasubramanian T (2014) | RCT  Level 2b | Coblator | 20 (NR) | 6.05 ± 1.36 (4-8) | NR | NR | Bleeding (ml) | **50.0 ± NR** | | |
|  |  |  |  |  |  |  |  |  |  |  |
|  |  | Curette | 20 (NR) | 6.20 ± 1.36 (4-8) |  |  | Bleeding (ml) | **20.0 ± NR** | | |
|  |  |  |  |  |  |  |  |  |  |  |
| Ozkiris M (2013) | RCT  Level 2b | Coblator | 30 (15 M; 15 F) | 5.79 ± 1.77 (4-8) | Adenoid hypertrophy | 41 | Operative time (min) | **20.5 ± 5.5*** | | |
|  |  |  |  |  |  |  | Bleeding (ml) | **5.25 ± 3.5*** | | |
|  |  |  |  |  |  |  | MCTT (mm/min) | 1.80 ± 0.21 | | **2.06 ± 0.31*** |
|  |  | Curette | 30 (16 M; 14 F) | 5.85 ± 1.83 (4-8) |  |  | Operative time (min) | **13.5 ± 5.2*** | | |
|  |  |  |  |  |  |  | Bleeding (ml) | **21.55 ± 8.2*** | | |
|  |  |  |  |  |  |  | MCTT (mm/min) | 1.33 ± 0.20 | | **1.35 ± 0.19*** |
| Di Rienzo L (2012) | Blinded RCT  Level 1b | Curette | 20 (9 M; 11 F) | 8.4 ± 3.42 (4-16) | Recurrent tonsillitis  tonsils degree >2 (Cassano) | 40 | RMN basal (pa/cm3 seg) | 1.22 ± 0.04 | | **0.60 ± 0.06*** |
|  |  |  |  |  |  |  | RMN decongestant (pa/cm3/seg) | 0.97 ± 0.02 | | **0.58 ± 0.06*** |
|  |  |  |  |  |  |  | adenoid grade (Cassano) | 3.3 ± 0.47 | | **1.8 ± 0.41*** |
|  |  |  |  |  |  |  | Bleeding (ml) | **30.7 ± 7.71*** | | |
|  |  | Coblator | 20 (9 M; 11 F) | 7.4 ± 3.39 (4-16) |  |  | RMN basal (pa/cm3/seg) | 1.31 ± 1.04 | | **0.33 ± 0.02*** |
|  |  |  |  |  |  |  | RMN decongestant (pa/cm3/seg) | 0.97 ± 0.02 | | **0.30 ± 0.02*** |
|  |  |  |  |  |  |  | Adenoid grade (Cassano) | 3.45 ± 0.51 | | **0.0 ± 0.0*** |
|  |  |  |  |  |  |  | Bleeding (ml) | **2.3 ± 2.32*** | | |
| Kim JW (2015) | Prospective cohort study  Level 2b | Coblator | 116 (M 72; F 44) | 6.2 ± 2.5 (NR) | Nasal obstruction, mouth breathing, sleep disordered breathing. Lateral radiograph with A/N ratio higher than 25% | 14 | Operation time (min) | **6.5 ± 2.7*** | | |
|  |  |  |  |  |  |  | Intraoperative bleeding (0-5) | **0-1 (77.6%) / 2 (18.1%) / 3 (4.3%) / 4 (0%) / 5 (0%)*** | | |
|  |  |  |  |  |  |  | Post-operative hemorrhage (*%*) | **0*** | | |
|  |  | MIcrodebrider + cauterization | 153 (M 94; F 59) | 6.6 ± 2.1 (NR) |  |  | Operation time (min) | **11.7 ± 4.1*** | | |
|  |  |  |  |  |  |  | Intraoperative bleeding (0-5) | **0-1 (17.6%) / 2 (26.8%) / 3 (53.0%) / 4 (2.6%) / 5 (0%)*** | | |
|  |  |  |  |  |  |  | Post-operative hemorrhage (*%*) | **0*** | | |
|  |  | Microdebrider alone | 119 (M 79; F40) | 6.9 ± 2.8 (NR) |  |  | Operation time (min) | **14.9 ± 5.2*** | | |
|  |  |  |  |  |  |  | Intraoperative bleeding (0-5) | **0-1 (5.0%) / 2 (50.4%) / 3 (38.7%) / 4 (5.9%) / 5 (0%)*** | | |
|  |  |  |  |  |  |  | Post-operative hemorrhage (*%*) | **3.36*** | | |
| Shapiro NL (2007) | Blinded RCT  Level 1b | Curette | 23 (M 15; F 8) | 6.1 ± NR  (2-16) | NR |  | Operation time (min) | **4.1 ± 0.5*** | | |
|  |  |  |  |  |  |  | Intraoperative bleeding (ml) | **<1(n=0) / 1-5 (n=3) / 5-10(n=9) / 10-15 (n=7) / 15-20(n=1) / 20-25(n=2) / 25-30(n=1)*** | | |
|  |  | Coblation | 23 (M 13; F 10) | 7.39 ± NR  (2-16) |  |  | Operation time (min) | **2.6 ± 0.6*** | | |
|  |  |  |  |  |  |  | Intraoperative bleeding (ml) | **<1(n=5) / 1-5 (n=14) / 5-10(n=3) / 10-15 (n=0) / 15-20(n=0) / 20-25(n=0) / 25-30(n=0)*** | | |
